# The potential value of the Purinergic pathway in the prognostic assessment and clinical application of kidney renal clear cell carcinoma

**DOI:** 10.18632/aging.205364

**Published:** 2024-01-04

**Authors:** Deqian Xie, Shijin Wang, Bowen Jiang, Guandu Li, Guangzhen Wu

**Affiliations:** 1 Department of Urology, The First Affiliated Hospital of Dalian Medical University, Dalian 116011, Liaoning, China

**Keywords:** Purinergic, KIRC, bioinformatics, machine learning, survival model

## Abstract

The Purinergic pathway is involved in a variety of important physiological processes in living organisms, and previous studies have shown that aberrant expression of the Purinergic pathway may contribute to the development of a variety of cancers, including kidney renal clear cell carcinoma (KIRC). The aim of this study was to delve into the Purinergic pathway in KIRC and to investigate its potential significance in prognostic assessment and clinical treatment. 33 genes associated with the Purinergic pathway were selected for pan-cancer analysis. Cluster analysis, targeted drug sensitivity analysis and immune cell infiltration analysis were applied to explore the mechanism of Purinergic pathway in KIRC. Using the machine learning process, we found that combining the Lasso+survivalSVM algorithm worked well for predicting survival accuracy in KIRC. We used LASSO regression to pinpoint nine Purinergic genes closely linked to KIRC, using them to create a survival model for KIRC. ROC survival curve was analyzed, and this survival model could effectively predict the survival rate of KIRC patients in the next 5, 7 and 10 years. Further univariate and multivariate Cox regression analyses revealed that age, grading, staging, and risk scores of KIRC patients were significantly associated with their prognostic survival and were identified as independent risk factors for prognosis. The nomogram tool developed through this study can help physicians accurately assess patient prognosis and provide guidance for developing treatment plans. The results of this study may bring new ideas for optimizing the prognostic assessment and therapeutic approaches for KIRC patients.

## INTRODUCTION

Kidney cancer is a relatively common malignant tumor of the urinary system and is one of the main tumors leading to significant mortality among patients [1]. The incidence of kidney cancer has been increasing in recent years. According to the American Cancer Society, about 80,000 patients are expected to be diagnosed with kidney cancer in 2023, and about 15,000 patients will die from the disease, which poses a great challenge to the global healthcare systems [2]. Kidney clear cell carcinoma (KIRC) is one of the more common subtypes of kidney cancer, accounting for 70-80% of new cases of kidney cancer. It has strong associations with geographical location, smoking, obesity, hypertension, and chronic kidney disease [3]. Currently, the main treatment options for KIRC include surgery, radiotherapy, targeted therapy, and immunotherapy [4]. However, because the symptoms of KIRC are not obvious in the early stages, patients often present to the clinic at an intermediate to late stage. Because KIRC is highly heterogeneous and has complex mechanisms, some KIRC patients are insensitive or resistant to treatment, resulting in a poor prognosis and low survival rate for KIRC patients [5, 6]. Therefore, exploring new therapeutic targets and prognostic markers to predict the survival and treatment response of KIRC patients and provide strong support for clinical decision-making is one of the important directions of current kidney cancer research.

The Purinergic pathway is a series of signaling processes mediated by extracellular purine nucleotides’ extreme derivatives (e.g., adenosine, ATP, and ADP) [7]. The purinergic pathway regulates immune processes, neurotransmitter release, apoptosis, and other important physiological processes in normal organisms by activating Purinergic receptors and related proteins on cell membranes [8]. However, abnormal changes in the Purinergic pathway may lead to the development of various diseases, including many cancers [9]. Recent studies have shown that tumor cells can use the Purinergic pathway to promote their proliferation, invasion, angiogenesis, and immune escape by increasing the production and release of purine nucleotide derivatives and altering the expression and sensitivity of Purinergic receptors [10]. At the same time, immune cells in the tumor microenvironment can also interact with tumor cells through the Purinergic pathway, thereby affecting tumor cell growth and metastasis [11]. It is noteworthy that Purinergic receptors are abundantly expressed in urological cancers, including KIRC, and that ATP can influence tumor-associated signaling pathways through the Purinergic receptor P2RX6, further promoting the migration and invasion of renal tumors [12, 13]. In addition, it has also been demonstrated that the Purinergic receptor P2RX7 is an independent poor prognostic indicator of postoperative survival specific to KIRC patients [14]. Based on these findings, we hypothesize that the Purinergic pathway plays a crucial role in the development and progression of KIRC, and further studies on the relationship between the Purinergic pathway and KIRC will help to gain insight into the pathogenesis of the disease and provide new ideas for the treatment and prognosis of KIRC patients.

In this study, 33 genes closely related to the Purinergic pathway (such as P2RY8, P2RX1, and GNAS) were selected, and their SNV, CNV, mRNA expression and methylation data were analyzed pan-cancer by bioinformatics methods. Meanwhile, we explored the relationship between these genes and the prognosis of KIRC patients and classical cancer pathways. To further reveal the mechanism of the Purinergic pathway in KIRC, we used cluster analysis to classify KIRC patients, and based on this, we conducted several studies, including histone modification genes, classical oncogene correlation analysis, targeted drug sensitivity analysis, and immune cell infiltration analysis. Finally, LASSO regression analysis was used to identify nine Purinergic genes (P2RY8, P2RX1, GNAS, P2RY11, ADORA2B, PANX1, ADORA1, P2RY6, and P2RY2) most closely related to KIRC, and a KIRC survival model was established based on these genes, which can effectively predict the survival of patients in the next few years and provide guidance for the diagnosis and treatment of KIRC. The results of this study are important for gaining insight into the Purinergic pathway in the development of KIRC and provide a solid basis for the treatment and management of the disease.

## MATERIALS AND METHODS

### Data acquisition and preliminary analysis

In the initial phase of the experiment, we selected 33 genes closely related to the Purinergic pathway in the “WikiPathways” dataset on the GSEA website (https://www.gsea-msigdb.org/) and performed a follow-up analysis of these genes [15]. The Cancer Genome Atlas (TCGA) is a public open-access database (https://portal.gdc.cancer.gov/) that contains genetic information on a wide range of human tumors and is extremely useful for single cancer types as well as for comprehensive pan-cancer analysis [16]. We downloaded genetic data of the Purinergic gene in 33 cancers from the TCGA database. We analyzed the downloaded data using Perl and R Studio for data analysis, and visualization using TBtools software to obtain single nucleotide variation (SNV) and mRNA expression differences of the Purinergic gene [17]. The GSCALite platform (http://bioinfo.life.hust.edu.cn/web/GSCALite/) can be used to analyze genomic variation, cancer pathway activity, and differential expression of genes in tumor patients versus normal subjects [18]. Based on the data obtained previously, we used the GSCALite platform to analyze the copy number variation (CNV) of Purinergic genes. We also examined the relationship between gene methylation levels, gene expression, and survival, as well as the extent of gene activation or repression of classical cellular pathways. We downloaded RNA-seq data of KIRC patients from the TCGA database via the R/Bioconductor package “TCGAbiolinks” in R Studio, which includes 72 normal and 539 tumor samples [19]. We also applied “TCGAbiolinks” to obtain clinicopathological information of KIRC patients, including tumor size (T), tumor metastasis status (M), tumor grade, tumor stage, age, survival status (fustat), and survival time (futime) [19]. For all statistical analyses conducted, a P-value of less than 0.05 was considered significant.

### Cluster analysis based on Purinergic scores

Based on the mRNA expression levels of Purinergic genes, we calculated the Purinergic score in R Studio using the R/Bioconductor package “GSVA” to quantify the expression of Purinergic genes [20]. Cluster analysis was performed based on the Purinergic score, and we generated a heat map of the results using the “pheatmap” package. We used the “ggpubr” package in R Studio to draw violin maps to show the expression differences between the three clustered samples. We also applied the “survival” package to plot the survival curves of the three clusters further to illustrate the effect of the three clusters on survival.

### Differential analysis of the three clustered samples in histone modification-related genes and classical oncogenes

To further clarify the possible regulatory mechanisms of Purinergic genes in tumorigenesis, we analyzed the expression of three clustered samples in three categories of genes closely related to tumorigenesis and visualized the results in the form of heat maps using the “pheatmap” package in R Studio. The three classes of genes closely related to tumorigenesis include 15 classical oncogenes such as MYC, KRAS and two classes of histone acetylation-related genes such as deacetylases (SIRT) and histone deacetylase inhibitors (HDACs). Classical oncogenes are a class of genes that promote tumor cell growth and regulate tumorigenesis through DNA mutations and epigenetic modifications [21]. SIRT has multiple isoforms, and different isoforms may have pro- or anti-oncogenic effects in different tumors. They can affect tumorigenesis by promoting DNA repair, pro-tumor metabolism, suppressing anti-tumor immunity, and other biological processes [22, 23]. Expression, mutation, or inappropriate recruitment of HDACs are found in various tumors, affecting the transcriptional activity of genes and influencing tumorigenesis through a range of biological pathways [24].

### Genomics-based sensitivity analysis of targeted drugs

We obtained drug sensitivity data between Purinergic genes and chemotherapeutic drugs from the Genomics of Drug Sensitivity in Cancer (GDSC) database (https://www.cancerrxgene.org/). The GDSC database contains information on the response and sensitivity of tumor cells to drugs and markers of drug response [25]. We screened 12 targeted drugs in the GDSC database. We constructed a ridge regression model in RStudio using the predictive function of the “pRRopheticl” package to estimate the drugs’ half-maximal inhibitory concentration (IC50) in three clustered samples. We used the “ggplot2” and “cowplot” software packages to draw box plots to visualize the IC50 of each drug in the three clustered samples. In addition, we explored the relationship between 25 Purinergic genes and GDSC drug sensitivity data using the GSCALite platform.

### Immune cell infiltration analysis of Purinergic gene

We used RStudio software and gene expression data from the TCGA database to quantitatively evaluate various immune cells using the single sample gene set enrichment analysis (ssGSEA) algorithm [26, 27]. We analyzed the correlation between Purinergic genes and immune cell infiltration levels using Spearman's rank correlation. We plotted bubble plots demonstrating the correlation coefficients between Purinergic scores and various immune infiltration metrics. In the bubble plots, the size of the spheres represents the strength of the correlation, while the color indicates the significance of the correlation. We found correlations between Purinergic scores and 29 indicators of immune infiltration. We used the R packages “ggplot2,” “dplyr,” and “data.table” “tidyr,” and “ggstatsplot” for data analysis and graphing. Then, we selected three representative indicators of immune infiltration: Parainflammation, CCR, and T-cell co-stimulation, and used the “ggdissterstats” package to plot scatter plots illustrating their correlation with the Purinergic score.

### KIRC prediction model development and validation

We applied the “Limma” package in RStudio to analyze the gene expression data of normal kidney and KIRC specimens in RNA-seq. The differences in Purinergic gene expression levels between KIRC and normal tissues were visualized using TBtools software. The co-expression relationships between any two Purinergic genes were established utilizing the “corrplot” package. Moreover, a univariate Cox regression analysis of the Purinergic genes was conducted, resulting in the identification of Purinergic genes exhibiting a P-value of <0.05. These genes were recognized as significantly correlated with the prognosis of KIRC patients. Concurrently, the calculated risk ratios in the analytical outcomes provided further insights into the pivotal role that Purinergic genes assume in the development of KIRC.

Based on a machine learning integration program, we have amalgamated 10 well-established machine learning algorithms, namely: CoxBoost, survival support vector machine (survivalSVM), Lasso, random survival forest (RSF), StepCox, supervised principal components (SuperPC), Ridge, partial least squares regression for Cox (plsRcox), elastic net (Enet), and generalised boosted regression modelling (GBM) [28, 29]. These 10 algorithms have been combined into 97 distinct algorithmic configurations, and predictive models have been established within the framework of Leave-One-Out Cross Validation (LOOCV). Further, we applied these constructed predictive models across three different cohorts to gauge their performance. The model's accuracy was quantified using the concordance index (C-index), with a higher C-index value denoting superior predictive accuracy.

Using the “glmnet” package in RStudio, we conducted a Lasso regression analysis to mitigate overfitting in gene expression data, aiming to simplify variables and optimize the model. Multivariate analysis helped identify the most potent Purinergic genes for the prognosis prediction of KIRC. Concurrently, we employed the Cox proportional hazards regression model to systematically evaluate the close correlation between the expression levels of Purinergic genes and patient survival across various cancer types. To quantify the risk, we computed a risk score for KIRC patients based on the product of gene expression levels and regression coefficients. During this process, we utilized the “survminer” package to determine the optimal cut-off value for the tumor group risk score, enabling effective risk stratification.

The formula for risk score computation is $\text{Risk}\;\text{score}=\sum_{\left(i=1\right)}^{n}\left[Exp_{i}*Coe_{i}\right]$.

Where n represents the number of genes, Exp_i_ denotes the expression level of the i^th^ gene, and Coe_i_ signifies the associated regression coefficient. Through these methods, we can evaluate the risk for KIRC patients based on gene expression data, and by using the median Purinergic score, we can classify samples into high-risk and low-risk groups. It provides robust support for further survival analysis and clinical prognostication.

Using the “survival” package in RStudio, we plotted the survival curves for the high-risk and low-risk groups. We identified the differences in survival between the two groups. We used the “survivalROC” package in RStudio to generate ROC curves for KIRC patients for the next 3, 5, 7, and 10 years. The “timeROC” package was then utilized to calculate the area under the curve (AUC) for each model. We used the “pheatmap” package to create heat maps to analyze the relationship between clinicopathological characteristics and Purinergic genes in two groups of KIRC patients. Univariate and multifactorial Cox regression analyses of clinical characteristics were performed using the “Survival” package to determine the correlation between patient age, grade, stage, T, M, risk score, and prognosis of KIRC patients. The nomogram drawn using the “rms” package allowed for a more convenient assessment of KIRC patients’ survival probability over the next 5, 7, and 10 years.

The UALCAN database (http://ualcan.path.uab.edu) explores, analyzes, and visualizes protein-coding gene expression data from 33 tumor types [30]. The Human Protein Atlas (HPA) database (https://www.proteinatlas.org/) includes protein expression from a wide range of tumor and normal tissues [31]. We obtained data on the differential protein expression of six Purinergic genes in normal versus KIRC tissues from the UALCAN database to further consolidate our findings. Immunohistochemical images of Purinergic genes P2RX7 and PANX1 in normal versus kidney cancer tissues were sourced from the HPA database, further supporting our findings.

### Data availability statements

All raw data from the study are included in the article/Supplementary Material and further inquiries can be directed to the authors.

## RESULTS

### Widespread genetic mutations and differential expression of Purinergic genes in multiple tumors

**Figure 1:**
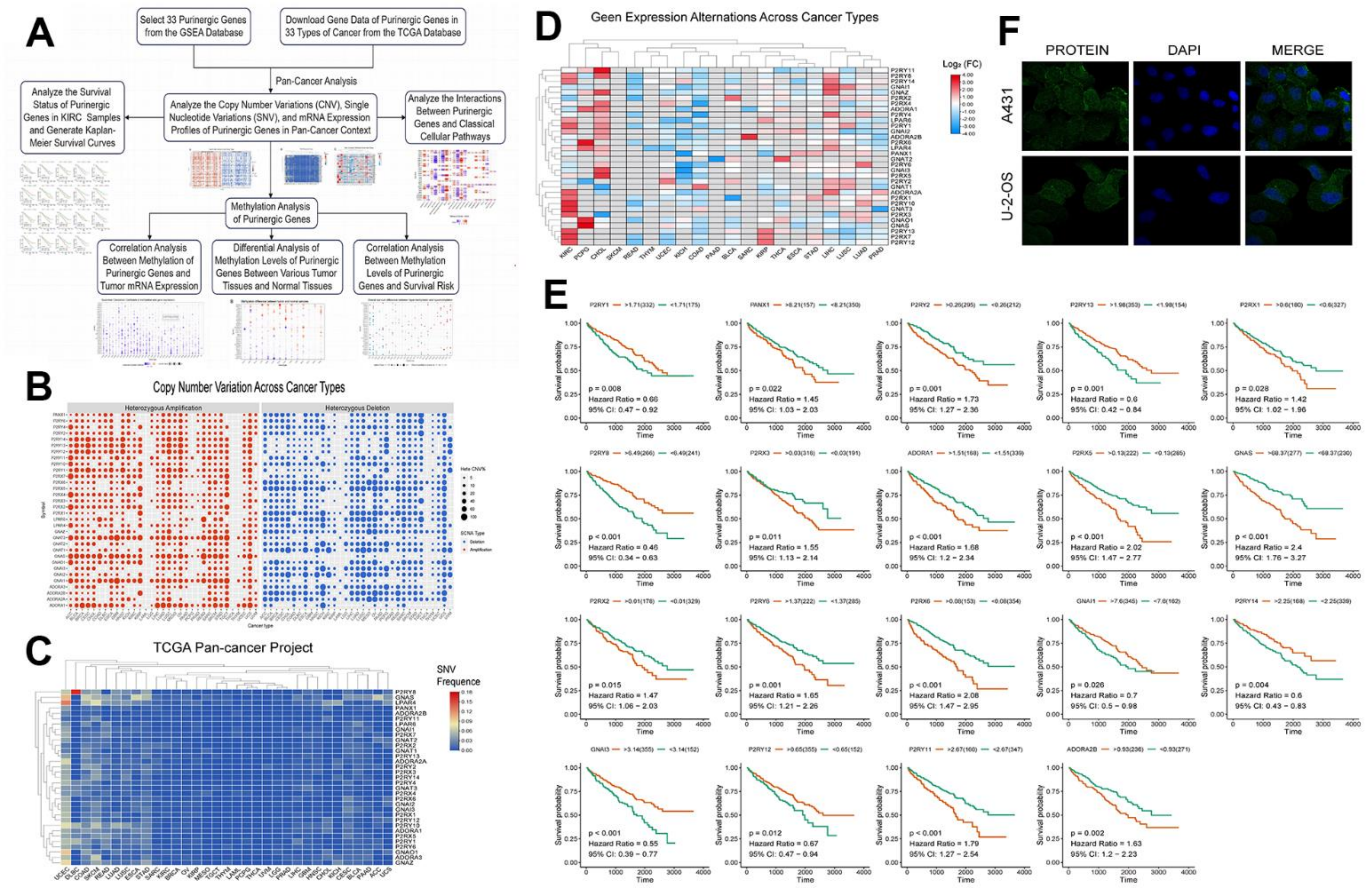
(**A**) We retrieved data on Purinergic genes and cancer patients from various databases, and conducted comprehensive analyses on the cancer associations and methylation patterns of these Purinergic genes. (**B**) CNV frequencies of 32 Purinergic genes in 33 cancer types. Red represents CNV gain, blue represents CNV loss, and the bubble size represents the degree of gain/ loss. (**C**) SNV frequencies of 33 Purinergic genes in 33 different cancer types. Red represents high mutation frequencies, and blue represents low mutation frequencies. (**D**) mRNA expression levels of 32 Purinergic genes in 20 different tumor types. Red represents increased mRNA expression, and blue represents decreased mRNA expression. (**E**) Survival curve analysis of the statistically significant Purinergic genes in KIRC samples. The names of Purinergic genes are labeled at the top of the curves. Orange represents the high expression of this Purinergic gene, and the green represents the low expression of this Purinergic gene. (**F**) The plot of immunofluorescence results of Purinergic gene PANX1 in A431 and U-2-OS cell lines from the HPA database.

To further investigate the differential expression and degree of genetic mutation of the Purinergic gene in multiple tumors, we performed a pan-cancer analysis based on the sample information provided by the TCGA database. The CNV frequencies (Figure 1B), SNV frequencies (Figure 1C), and mRNA expression profiles (Figure 1D) of Purinergic genes in multiple tumors were obtained. CNV results showed that Purinergic genes *P2RY12*, *P2RY13*, *P2RY14*, *P2RY1*, *P2RX2*, *P2RX4*, *P2RX7*, *GNAT3*, *GNAS*, *GNAI1*, and *ADORA1* showed gain in 33 different types of cancers. *PANX1, P2RY2, P2RY4, P2RY6, P2RX1, P2RX5, P2RX6, LPAR6, GNAZ, ADORA2A, ADORA2B* showed a gain in 33 different types of cancer showed CNV deletion. Purinergic genes were present in 33 tumors with varying degrees of SNV, with Purinergic genes having the highest rate of SNV mutations in UCEC. mRNA expression profiles of Purinergic genes demonstrated the expression levels of these genes in 20 tumors. Among them, the greatest differences in mRNA expression of these genes were observed in KIRC. Most of the genes showed significantly higher mRNA expression in KIRC than in normal tissues, including *P2RY12, P2RX7, P2RY13, P2RX3, GNAT3, P2RY10, P2RX1, ADORA2A, GNAI2, P2RY1, LPAR6, P2RY8, P2RY14*. In addition, many Purinergic genes were significantly less expressed in KIRC than in normal tissues, including *GNAI1, P2RX2, ADORA1, PANX1, P2RY6, GNAI3*, and *P2RY2*.

Figure 1E further delves into the survival of Purinergic genes in KIRC samples, and the results of the analysis show that among the 19 Purinergic genes covered, the high expression of 12 Purinergic genes showed a negative correlation with the prognosis of KIRC patients. Particularly noteworthy were four Purinergic genes, *P2RX5, GNAS, P2RX6*, and *P2RY11*, which showed a significant association between their high expression in KIRC patients and poor patient prognosis (P < 0.001). In contrast, the results of the study also revealed a positive correlation between the high expression of five genes, *P2RY13, P2RY8, P2RY14, P2RY12*, and *GNAI3*, and the prognosis of KIRC patients. In addition, to gain a deeper understanding of Purinergic gene expression in a variety of tumor cells, and based on the immunofluorescence images obtained from the HPA website (Figure 1F), we found that the Purinergic gene *PANX1* was significantly expressed in the cell cytoplasm in both A431 cell line (Human epidermoid carcinoma cells) and U-2-OS cell line (Human osteosarcoma cells).

### Linkage of methylation analysis of Purinergic genes to classical pathways

**Figure 2:**
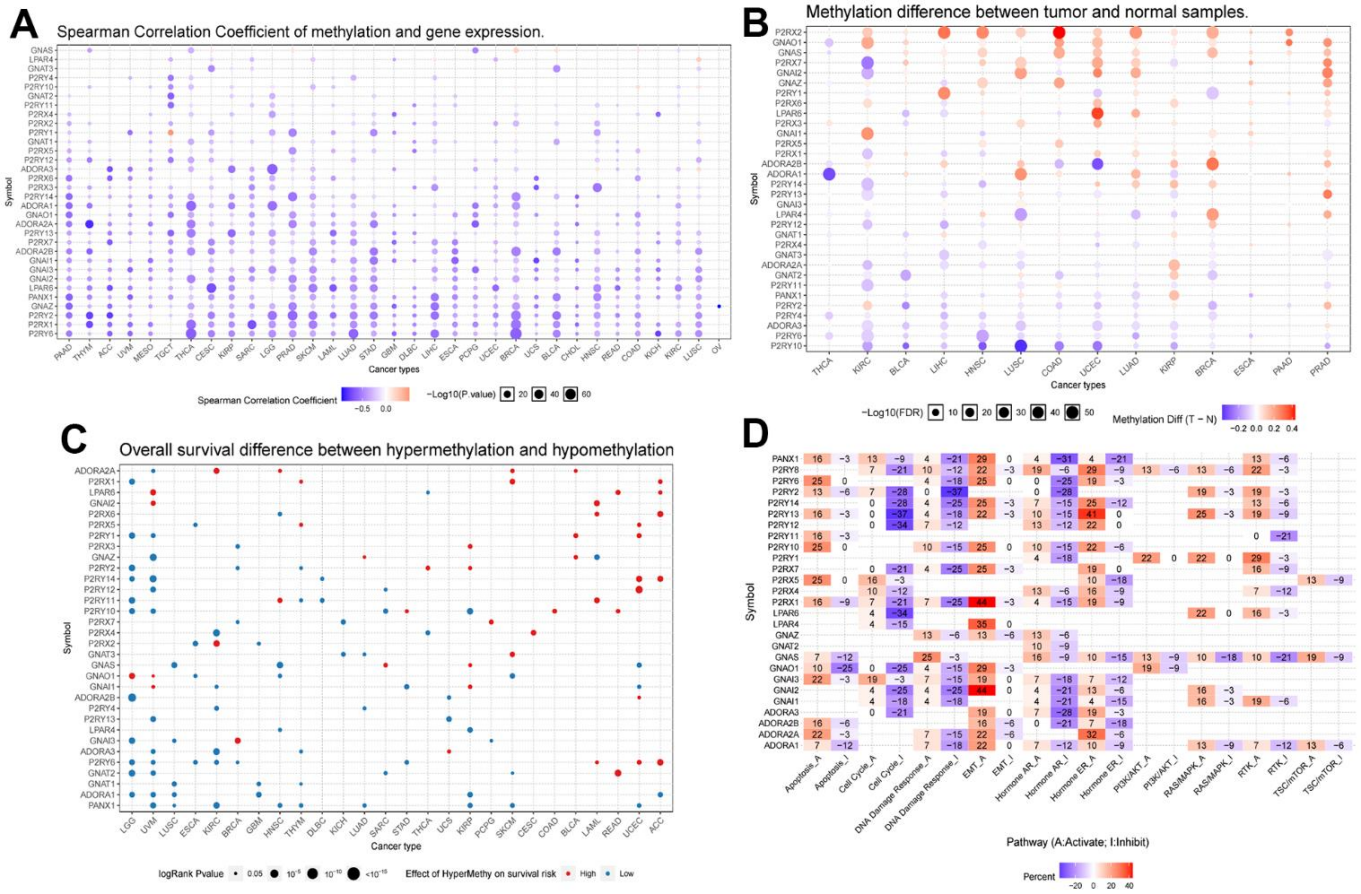
(**A**) Correlation analysis between Purinergic gene methylation and mRNA expression. The color bar indicates the correlation coefficient's magnitude, and the dots' size represents the comparison between the P-value and 0.05. (**B**) Analysis of the difference in the degree of methylation of the Purinergic gene in different tumor tissues and normal tissues. The color bar indicates the degree of difference, and the size of the dots represents the comparison between the P-value and 0.05. (**C**) Correlation analysis between the degree of methylation of the Purinergic gene and survival risk. Red dots represent high survival risk, blue dots represent low survival risk, and the size of the dots represents the comparison between the P-value and 0.05. (**D**) Relationship between Purinergic genes and classical cellular pathways. A represents activation, and I represent suppression.

DNA methylation is an epigenetic regulatory mechanism that can affect the transcriptional potential of genes [32]. Aberrant DNA methylation can lead to silencing or activating tumor-associated genes, promoting tumorigenesis, maintenance, and progression. In addition, methylation changes can also affect tumor immune infiltration and therapeutic response [33]. We obtained data on Purinergic gene methylation in pan-cancer in the TCGA database and analyzed the data using the GSCALite platform. The analysis showed that the expression of Purinergic genes *P2RY6, P2RX1*, and *P2RY2* in multiple tumors strongly correlated with the level of methylation (Figure 2A). The methylation levels of Purinergic genes in multiple tumors were very different compared to normal samples, especially in KIRC (Figure 2B). We further investigated the association between Purinergic genes and the survival of KIRC patients. We showed that high methylation levels of Purinergic genes *ADORA2A, P2RX4, P2RX2, GNAI1, P2RY4, ADORA3, P2RY6, PANX1* had an impact on the overall survival of KIRC patients (Figure 2C). Tumor development is usually closely linked to various classical biological pathways that play important roles in regulating inflammatory responses, cell proliferation, apoptosis, angiogenesis, and immune escape, affecting the course of tumorigenesis [34]. To gain a deeper understanding of the role of Purinergic genes in tumor development, we studied their classical signaling pathways. Our research reveals that Purinergic genes play regulatory roles in multiple biological processes, involving mechanisms such as cell apoptosis, cell cycle, DNA damage response, and epithelial-mesenchymal transition (EMT). Specifically, the Purinergic genes *P2RX1* and *GNAI2* have significant roles in promoting the EMT process of tumor cells, while *P2RY13* can inhibit the progression of the tumor cell cycle. Additionally, *P2RY2* demonstrates its key regulatory capacity in suppressing the DNA damage response (Figure 2D).

### Cluster analysis based on Purinergic gene scores

**Figure 3:**
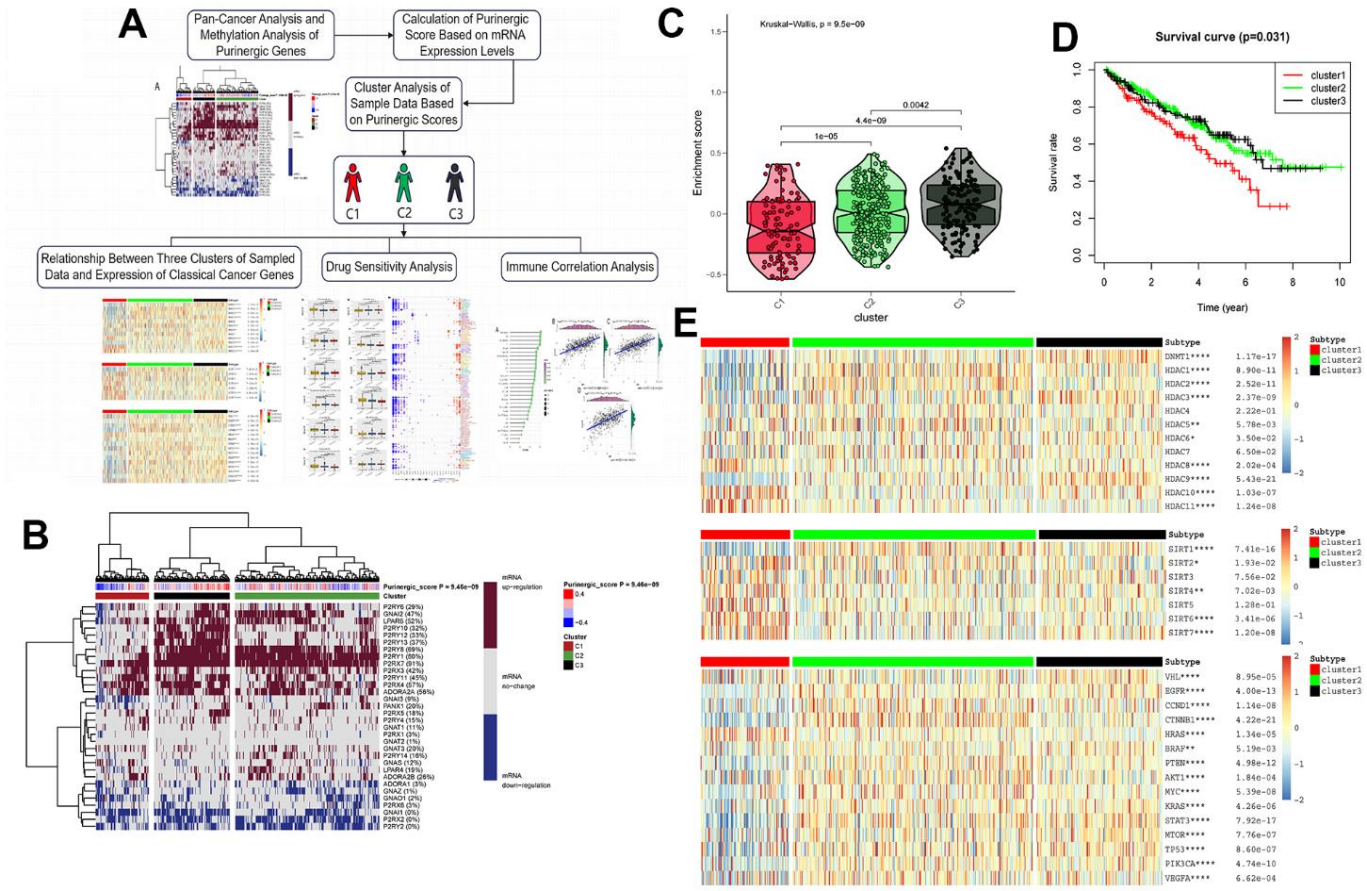
(**A**) Upon finalizing the pan-cancer and methylation studies of the Purinergic gene, we embarked on a clustering analysis of all data samples, grounded on the mRNA expression profiles of this gene. The clustering segmented the samples into three distinct groups. Subsequent analyses of these groups encompassed canonical oncogene studies, drug sensitivity evaluations, and immune infiltration assessments. (**B**) Purinergic scores were calculated based on the level of mRNA expression of Purinergic genes. KIRC samples were divided into three groups according to different levels of Purinergic scores: low expression group (cluster 1), normal expression group (cluster 2), and high expression group (cluster 3). The brown color in the right color bar indicates increased mRNA expression, the gray color indicates no change in mRNA expression, and the blue color indicates decreased mRNA expression. The closer the Purinergic score is to 0.4, the redder the color is, and the closer it is to -0.4, the bluer the color is. The KIRC samples were divided into three groups by cluster analysis; red, green, and black represent cluster 1, cluster 2, and cluster 3, respectively. (**C**) The violin plot shows the enrichment scores of the three clustered samples, the results show C3 > C2 > C1, and the p-values for comparison between groups are shown in the figure. (**D**) Survival curve analysis of the three clustered samples. The results show that the survival rate of KIRC patients in the C1 group is much lower than that of KIRC patients in the C2 and C3 groups. Red represents the C1 group, green represents the C2 group, and black represents the C3 group. The horizontal coordinate unit is the number of years of survival, and the vertical coordinate unit is the probability of survival. (**E**) Heat map showing the association between the three clustered samples and HDAC, SIRT, and classical oncogenes expression, respectively. The color bar red represents high expression, and blue represents low expression. In the legend, red represents cluster 1, green represents cluster 2, and black represents cluster 3.

Based on the Purinergic gene mRNA expression, we used cluster analysis to classify the KIRC patient samples obtained from the TCGA database, with Purinergic scores representing different mRNA levels (Figure 3B). Based on the differences in Purinergic gene expression, we obtained three clustered samples: Cluster1 (C1), Cluster2 (C2), and Cluster3 (C3). C1 represents the low expression of Purinergic genes, C2 represents the regular expression of Purinergic genes, and C3 represents the high expression of Purinergic genes. The violin plot visualizes the degree of difference between the three clustering groups, ranked by enrichment score as C3 > C2 > C1 (Figure 3C). The P-value for the difference between the three clustering groups was <0.05, which was statistically significant. Kaplan-Meier survival analysis was performed on samples from the three clustered groups, and the analysis showed that the difference in Purinergic gene expression was closely related to the prognosis of patients. Compared with the prognosis of KIRC patients in groups C2, and C3, the prognosis of KIRC patients was the worst in group C1 with low expression of the Purinergic gene (Figure 3D).

### Expression of classical oncogenes and histone modification-related genes in three clustered samples

We analyzed the expression of two major histone modification genes, HDAC and SIRT, in three different clusters (Figure 3E). The study clearly indicated that the expression levels of *HDAC8, HDAC10*, and *HDAC11* were significantly increased within the C1 group. In contrast, the expression of HDAC family genes such as *HDAC1, HDAC2, HDAC3*, and *HDAC9* was significantly lower in the C1 group than their levels in the C2 and C3 groups. The SIRT family genes showed similar expression patterns, for example, the expression of *SIRT1* in the C1 group was significantly lower than its levels in the C2 and C3 groups, whereas *SIRT2, SIRT4, SIRT6*, and *SIRT7* were more highly expressed in the C1 group. These aberrant expressions of HDAC and SIRT family genes suggest that the differences in Purinergic gene expression in tumor cells are strongly associated with histone modification processes.

Several previous clinical studies have reported that selective HDAC inhibitors have demonstrated encouraging clinical effects in the treatment of KIRC, as well as relatively mild and short-duration adverse effects [35–37]. However, for some KIRC patients, the clinical use of HDAC inhibitors did not significantly improve their clinical outcomes [38]. Our findings may provide a further answer for this. Considering the differences in the expression levels of Purinergic genes among KIRC patients, selecting specific HDAC inhibitors based on the expression of these genes might provide more precise treatment for patients. For example, in KIRC patients with low expression of Purinergic genes, the use of HDAC inhibitors targeting *HDAC8, HDAC10*, and *HDAC11* may help to inhibit the invasive and metastatic ability of tumor cells, which is closely associated with a good prognosis for patients. In addition, it has been noted that *SIRT1* is significantly and positively correlated with VEGF expression, and *SIRT1* can promote tumor angiogenesis and create a favorable environment for tumor growth [39]. In our study, KIRC patients with high expression of the Purinergic gene showed higher levels of *SIRT1* expression, and increased VEGF expression may be one of the reasons for the poor prognosis of such patients. Therefore, in clinical treatment, the application of *SIRT1* inhibitors for this group of patients may improve the therapeutic effect, but more clinical trials are still needed to support this conclusion.

We further focused on the expression of classical oncogenes in the three clustered samples, and the analysis showed that the three clustered samples were closely associated with all classical oncogenes. Except for HRAS, the expression levels of all the classical oncogenes were generally lower in the C1 group and higher in the C3 group. Compared with the expression levels of HRAS in the C2 and C3 groups, the expression levels of HRAS were higher in the C1 group. The high expression of classical oncogenes *EGFR, TP53*, and *KRAS* in the C3 group may suggest that targeting these classical oncogenes in KIRC patients with high expression of Purinergic genes may lead to a positive effect on tumor treatment (Figure 3E).

### Correlation analysis of Purinergic score and related genes with drug sensitivity

**Figure 4:**
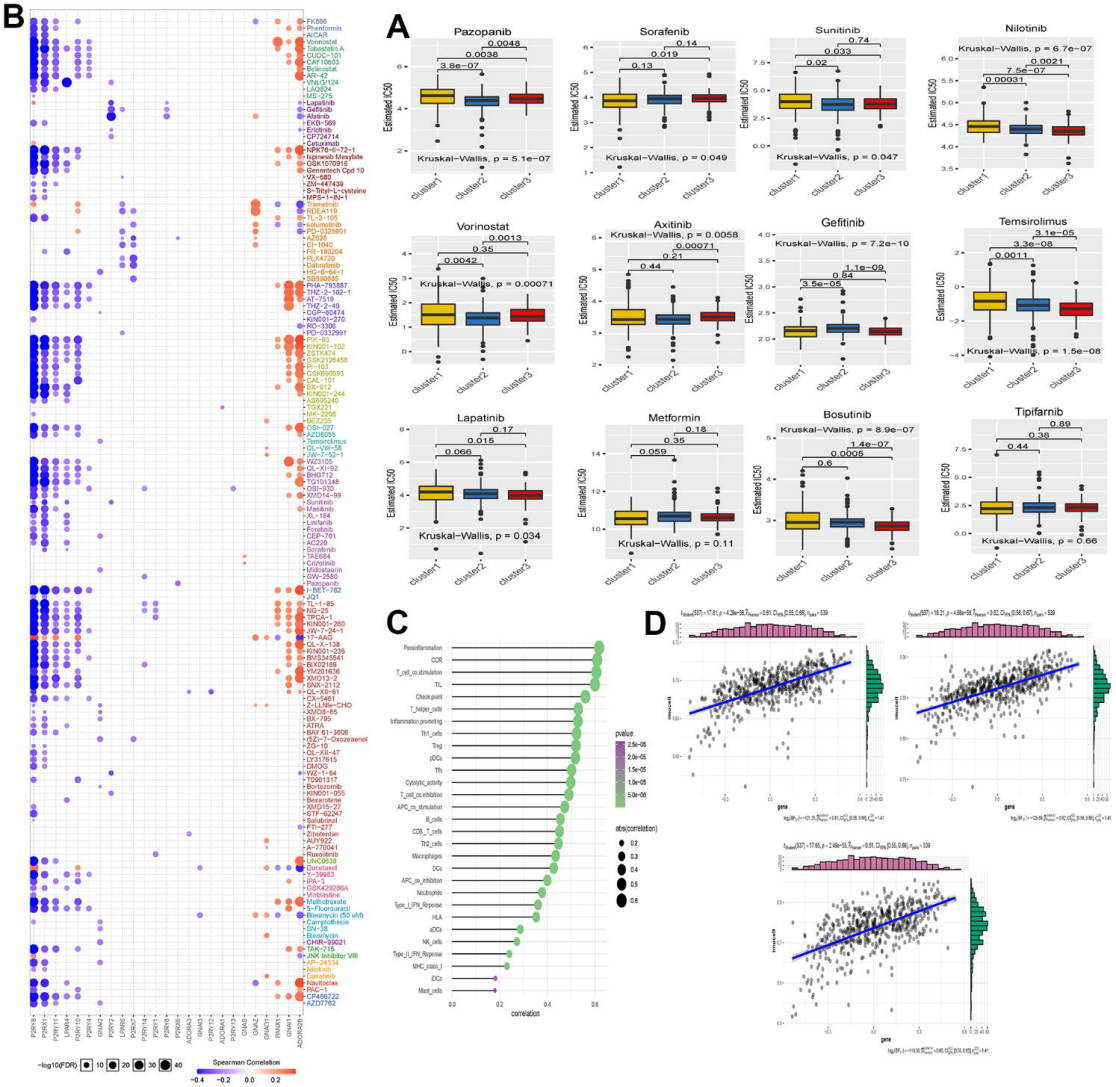
(**A**) The box plot shows the IC50 prediction analysis of the three clustered samples with commonly used KIRC-targeted drugs. The names of the targeted drugs are shown at the top of the box line plot, and the p-values for the group comparisons are shown in the box line plot. (**B**) Heat map showing correlation analysis between drug sensitivity data obtained from the GDSC database and mRNA expression levels of Purinergic genes. (**C**) Bubble diagram showing the degree of correlation between Purinergic and immune infiltrating factors. The size of the bubbles indicates the level of correlation between the two, and the color bar indicates the size of the P**-**value. (**D**) The three scatter plots show the correlation between Purinergic and Parainflammation, CCR, and T-cell-co.stimulation, respectively.

To further explore the potential value of the Purinergic pathway in the clinical management of KIRC patients, we selected 12 commonly used targeted drugs from the GDSC database and analyzed the effect of the Purinergic score on the IC50 of these targeted drugs, with lower IC50 values representing higher drug sensitivity (Figure 4A). The results of the drug IC50 prediction analysis showed that the IC50 values of Tipifarnib, Metformin, and Axitinib among the 12 commonly used targeted drugs did not differ significantly among the three groups, indicating that the difference in Purinergic did not have a significant effect on the sensitivity of these three drugs. The IC50 values of Vorinostat in group C2 were significantly lower compared to the IC50 values of this targeted agent in groups C1 and C3, suggesting that this drug could be used in treating KIRC patients with normal Purinergic. On the contrary, the IC50 value of Gefitinib in group C2 was significantly higher compared to the IC50 values of this drug in groups C1 and C3, indicating that this drug is less sensitive for patients with Purinergic normal KIRC and should be avoided during treatment. In addition, the IC50 predictions for most of the targeted drugs in the C3 group were significantly lower than those in the C1 group, including Pazopanib, Sunitinib, Nilotinib, Temsirolimus, Lapatinib, Bosutinib, indicating that patients with high expression of the Purinergic gene KIRC patients with high expression of Purinergic gene are more sensitive to these targeted drugs and their use in this group of patients may lead to better results. Specifically, the IC50 predictions for the targeted drug Sorafenib in the C1 group were significantly lower than those in the C3 group. It may indicate that this drug is more effective in treating KIRC patients with low Purinergic gene expression. We also analyzed GDSC and Purinergic gene mRNA expression and drug response data on the GSEA platform, and the results showed that most Purinergic gene mRNA expression correlated strongly with GDSC drug sensitivity. For example, *P2RY8* and *P2RX1* were negatively correlated with the sensitivity of various targeted drugs, and *GNAI1* and *ADORA2B* were positively correlated with the sensitivity of various targeted drugs (Figure 4B). This series of results suggests that the Purinergic pathway may lead to new ideas and more precise directional guidance for developing and applying future KIRC-targeted drugs.

### Correlation analysis of Purinergic pathway and immune cell infiltration

Immunotherapy of tumors has nowadays become a very important part of tumor treatment, and immunotherapy of tumors is closely related to the regulation of the tumor microenvironment, and different types and states of immune cells in the tumor microenvironment also affect tumor development and prognosis and influence the response of tumor cells to immunotherapy [40, 41]. To further investigate the potential value of the Purinergic pathway in the course of KIRC immunotherapy, we analyzed the correlation between the Purinergic pathway and immune cell infiltration. The bubble plots demonstrate the correlation between immune cell infiltration indicators and the Purinergic pathway (Figure 4C). We selected three immunocyte infiltration indices, namely Parainflammation, Chemokine Receptor (CCR), and T-cell co-stimulation, which are statistically significant. These indices were represented in a scatter plot to demonstrate the correlation with the Purinergic pathway. The analysis findings align with the results presented in the bubble chart, affirming a positive correlation between these three immunocyte infiltration indices and the Purinergic pathway (Figure 4D).

### Differential expression of Purinergic genes in KIRC samples and prognostic analysis

**Figure 5:**
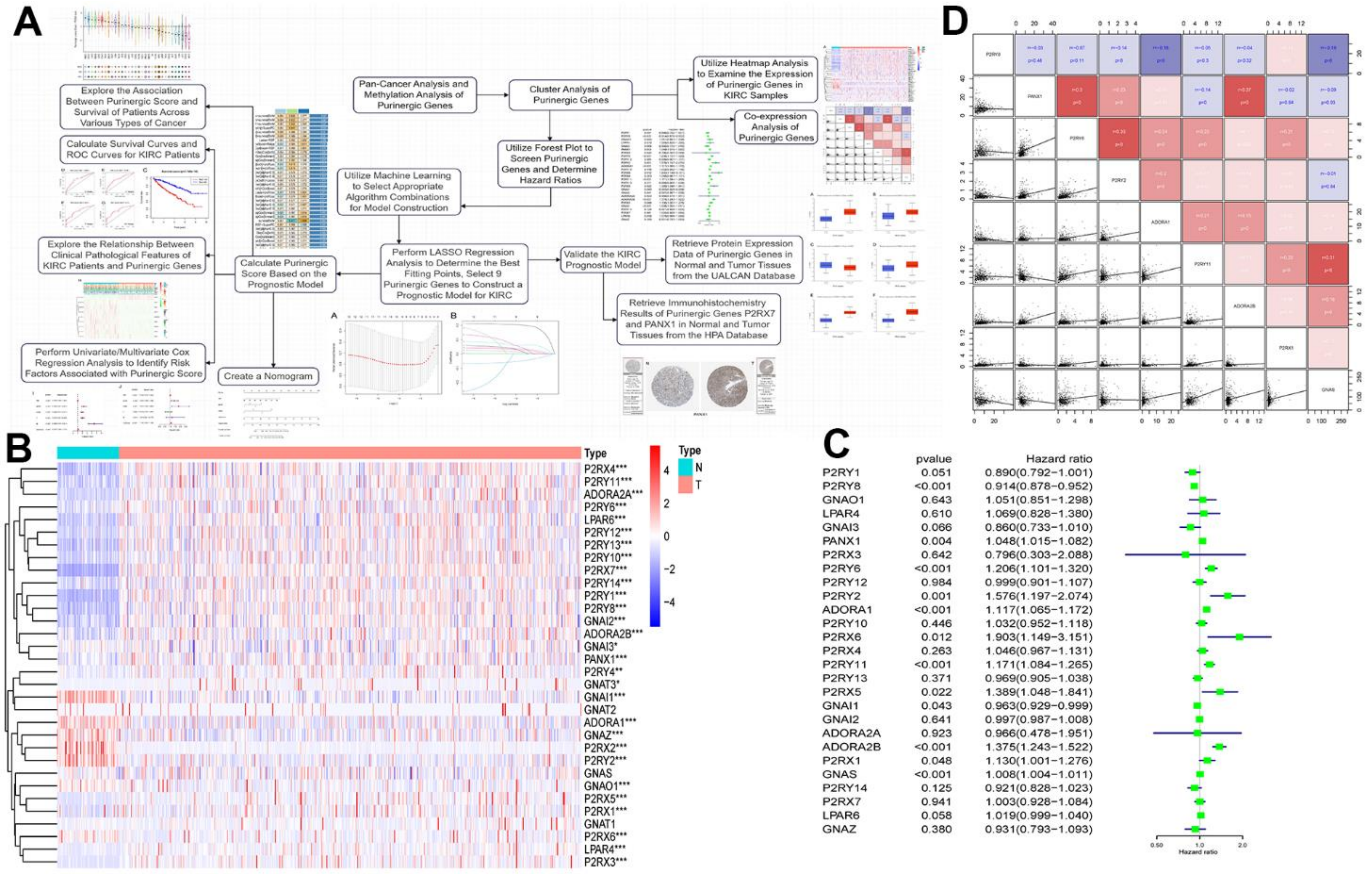
(**A**) Upon completing the initial analysis, we further explored the function and divergence of the Purinergic gene in KIRC. Using integrated machine learning techniques, we selected the most suitable algorithmic blend to construct a KIRC prognostic model based on the Purinergic gene. Rigorous validation was performed to ensure the model's precision. (**B**) Heat map showing the difference in Purinergic gene expression in KIRC tissue versus normal kidney tissue. The light blue color in the legend represents normal kidney tissue, and the light red color represents KIRC tissue. Red in the color bar indicates increased Purinergic gene expression, and blue indicates decreased Purinergic gene expression. * indicates P < 0.05, ** indicates P < 0.01, and *** indicates P < 0.001. (**C**) Forest plot showing 95% confidence intervals and risk ratio analysis for different Purinergic genes in KIRC. (**D**) Co-expression analysis between the nine Purinergic genes. The scatter plot represents the regression relationship between two Purinergic genes, and the correlation coefficients between two Purinergic genes are distinguished by color, with red indicating a positive correlation, blue indicating a negative correlation, and darker color indicating a stronger correlation.

We analyzed RNA sequencing data from 539 KIRC patients and 72 normal kidney samples, sourced from the TCGA database. The analysis showed that 29 Purinergic genes out of 32 Purinergic genes significantly differed between the two groups. Most of the Purinergic genes increased expression in tumor tissues compared to normal kidney tissues. Specifically, the expression of five Purinergic genes, *GNAI1, ADORA1, GNAZ, P2RX2,* and *P2RY2,* was significantly lower in KIRC tissues than in normal tissues (Figure 5B). To further explore the effect of Purinergic genes on analysis (Figure 5C). The analysis showed that 12 out of 27 Purinergic genes were able to affect the prognosis of KIRC patients significantly. Among them, *PANX1, P2RY6, P2RY2, ADORA1, P2RX6, P2RY11, P2RX5, ADORA2B, P2RX1, GNAS* were risk factors (hazard ratio >1) and *P2RY8, GNAI1* were protective factors (hazard ratio <1). We selected nine Purinergic genes with statistically significant (p < 0.05) effects on KIRC for co-expression analysis. The results showed strong co-expression relationships between these genes, with positive correlations between most of the Purinergic genes and negative relationships between only a small number of Purinergic genes (Figure 5D).

### Construction and validation of the Purinergic gene-based KIRC survival model

**Figure 6:**
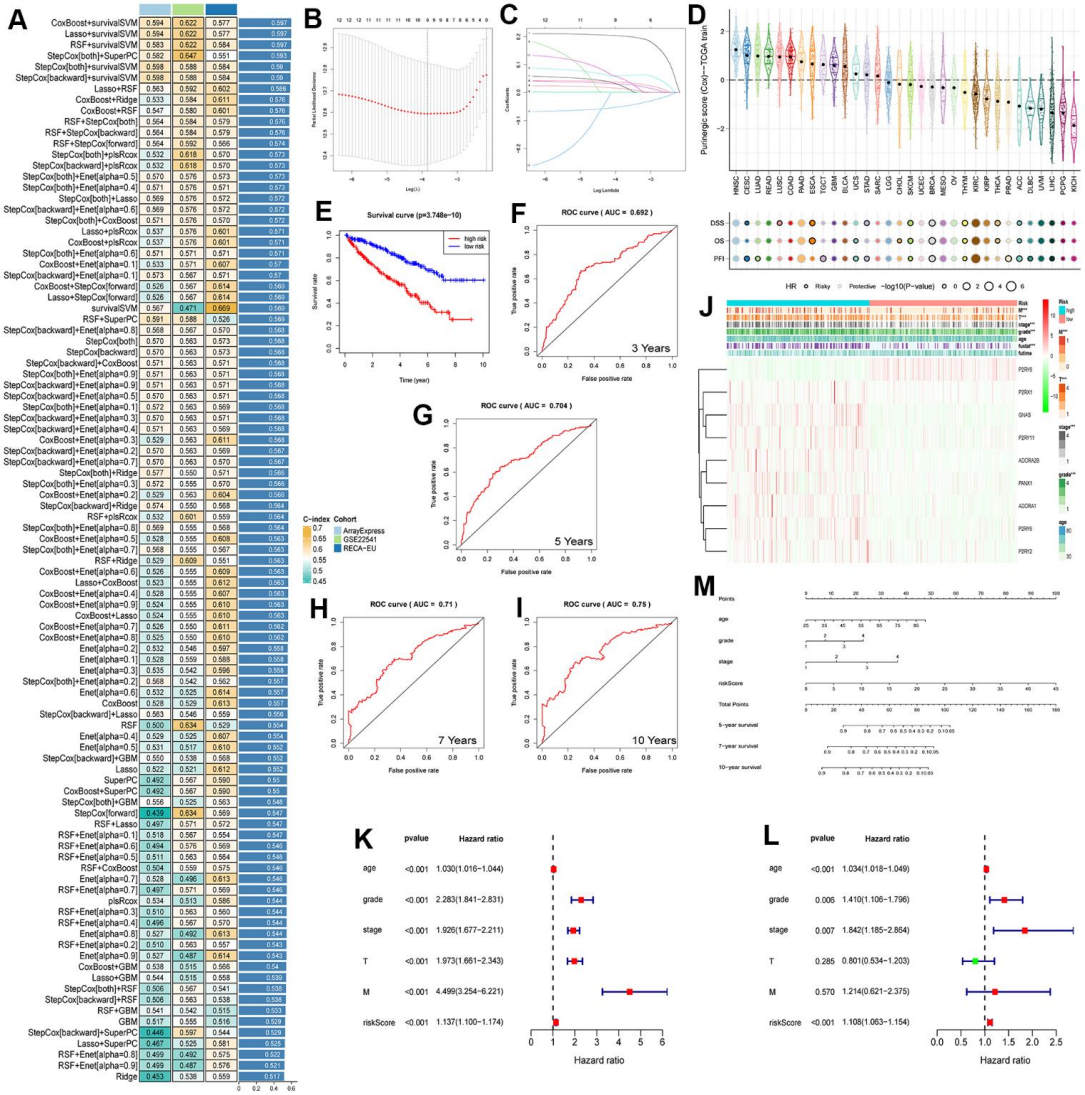
(**A**) The performance of 97 prediction models, developed using the LOOCV framework, as evaluated by their C-index across three distinct datasets. (**B**, **C**) KIRC survival models were established by LASSO regression analysis identifying 9 Purinergic genes. (**D**) The correlation between Purinergic gene expression levels in pan-cancer and the survival outcomes of patients. (**E**) KIRC patients were divided into high-risk and low-risk groups according to the median risk score, and survival analysis was performed for both groups. (**F**–**I**) ROC survival curve analysis was performed on the established KIRC model to verify the accuracy of the survival model. The AUC values for the next 3, 5, 7, and 10 years were 0.692, 0.704, 0.71, and 0.75, respectively, and an AUC greater than 0.7 is usually considered predictive. (**J**) Heat map demonstrating the association between Purinergic gene expression and clinicopathological features of KIRC in the high-risk versus low-risk groups of KIRC. Light blue represents the KIRC high-risk group, and light red represents the KIRC low-risk group. Red in the color bar indicates an increase in Purinergic gene expression, and green indicates a decrease in Purinergic gene expression. *** indicates P < 0.001. (**K**, **L**) Univariate and multifactorial Cox regression analysis between risk scores, clinicopathological characteristics, and overall survival in KIRC patients. (**M**) A nomogram based on the Purinergic gene-associated KIRC survival model can be used to calculate the survival risk of KIRC patients for the next 5, 7, and 10 years by quantifying various factors in KIRC patients.

Upon conducting a univariate Cox regression analysis on 27 Purinergic genes, we identified 12 Purinergic genes significantly associated with the prognosis of KIRC (P < 0.05) (Figure 5C). Utilizing a machine learning integration program, we fed the data from these 12 genes as input features into our machine learning workflow for in-depth analysis. Specifically, we employed 10 classic machine learning algorithms, combining them into 97 unique algorithm configurations. Predictive models were then constructed within the LOOCV framework. To evaluate the prediction accuracy of each model, we computed the C-index for these 97 predictive models on the provided dataset, ranking their accuracy based on the C-index values. Notably, our results revealed that among the 97 predictive models, the combination of Lasso+survivalSVM had a C-index of 0.597, showcasing superior predictive accuracy (Figure 6A). Within the LASSO regression, by observing the intersection of the vertical line and the curve, we determined the optimal fitting point, which is the number of selected genes. Based on the minimization criterion, we further narrowed down to 9 key Purinergic genes, forming a risk signature model. These genes include *P2RY8, P2RX1, GNAS, P2RY11, ADORA2B, PANX1, ADORA1, P2RY6*, and *P2RY2* (Figure 6B, 6C).

Leveraging this risk signature model, we computed the Purinergic scores for various cancers and delved deep into the correlation between Purinergic scores and survival. The study findings unveiled that in Head and Neck Squamous Cell Carcinoma (HNSC) and Lung Adenocarcinoma (LUAD), Purinergic scores were markedly elevated. However, in Pheochromocytoma and Paraganglioma (PCPG) and Kidney Chromophobe (KICH), the scores were lower. Additionally, levels of Purinergic scores were closely tied with the survival of cancer patients. We assessed survival outcomes using metrics like Disease-Specific Survival (DSS), Overall Survival (OS), and Progression-Free Interval (PFI). High Purinergic scores are often linked with poor prognostic outcomes in several cancers, whereas low scores might indicate favorable prognosis for most cancer patients. Nonetheless, for cancers like Melanoma (SKCM), Breast Invasive Carcinoma (BRCA), and Thymoma (THYM), a low Purinergic score might result in unfavorable outcomes (Figure 6D). Based on the median Purinergic score in KIRC patients, we categorized samples into low-risk and high-risk groups. Survival analysis was then conducted using Kaplan-Meier survival curves (K-M curves). This analysis vividly indicated that the overall survival rate for high-risk patients was significantly lower than for those in the low-risk group (Figure 6E), aligning with our pan-cancer analysis results.

To further appraise the predictive ability of our KIRC survival model, we employed ROC curve analysis to evaluate the AUC scores for KIRC patients at the 3, 5, 7, and 10-year marks (Figure 6F–6I). The 3-year AUC was 0.692, the 5-year AUC was 0.704, the 7-year AUC was 0.71, and the 10-year AUC was 0.75. An AUC greater than 0.7 is usually considered predictive, so the KIRC survival model constructed based on the Purinergic gene has a high predictive value for the prognosis of KIRC patients in the next 5, 7, and 10 years.

To further explore the relationship between the clinicopathological features of KIRC patients and Purinergic genes, we visualized the correlation between nine Purinergic genes and clinicopathological features of KIRC patients in a heat map (Figure 6J). The analysis showed that the expression of eight Purinergic genes, *P2RX1, GNAS, P2RY11, ADORA2B, PANX1, ADORA1, P2RY6, P2RY2*, was significantly increased, and the expression of *P2RY8* was significantly decreased in the high-risk group of patients. The KIRC survival model we established was closely related to the tumor's M, T, grade, stage, and fustat. Meanwhile, one-way Cox regression analysis showed that age, grade, stage, T, M, and risk score were associated with the OS of KIRC patients (Figure 6K). Multi-factor Cox regression analysis showed that age, grade, stage, and risk score were independent risk factors for prognosis in KIRC patients (Figure 6L). We created a nomogram for predicting risk in KIRC patients, with scores in the first row and age, tumor grade, tumor stage, and risk score in rows 2-5, respectively. The total score in the sixth row was obtained by summing the scores corresponding to rows 2-5. Based on the total score in the sixth row, we could easily predict the survival rate of KIRC patients in the next 5, 7, and 10 years (Figure 6M).

**Figure 7:**
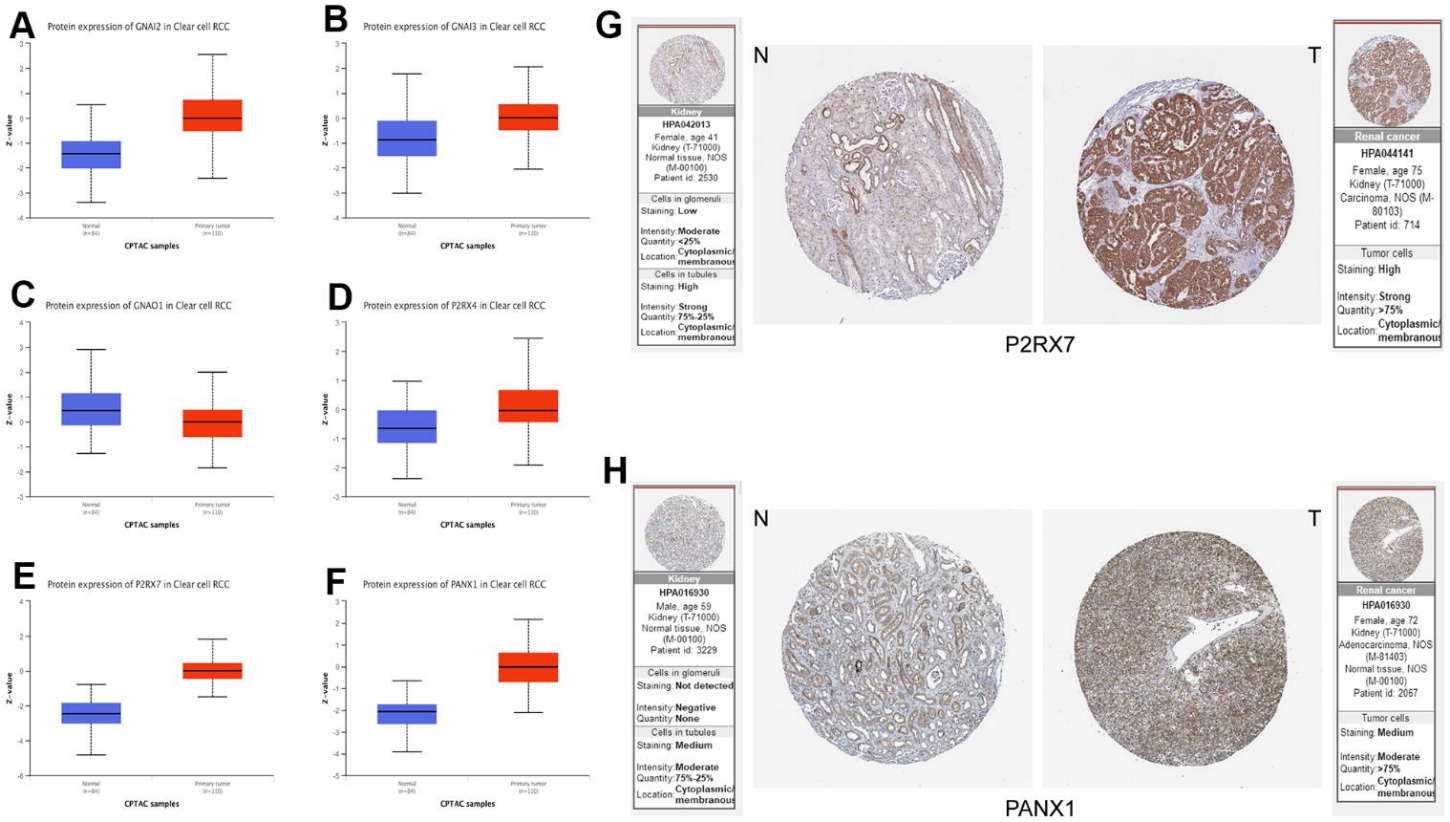
(**A**–**F**) Protein expression data of Purinergic genes GNAI2, GNAI3, GNAO1, P2RX4, P2RX7, PANX1 in normal tissues vs. KIRC tissues from UALCAN database. (**G**, **H**) Immunohistochemical images of Purinergic genes P2RX7 and PANX1 in normal tissues compared with kidney cancer tissues in the HPA database.

Finally, to verify the accuracy and validity of our constructed model, we performed a series of complementary experiments to validate its reliability. We obtained the protein expression data of Purinergic genes *GNAI2, GNAI3, GNAO1, P2RX4, P2RX7*, and *PANX1* in normal tissues and tumor tissues in the UALCAN database. The analysis results showed that the expression levels of Purinergic genes GNAI2, GNAI3, P2RX4, P2RX7, and PANX1 were significantly higher in tumor tissues than in normal tissues, consistent with the mRNA expression results (Figure 7A–7F). In addition, we also downloaded the immunohistochemical results of Purinergic genes *P2RX7* and *PANX1* in normal and tumor tissues through the Human Protein Atlas (HPA) website (http://www.proteinatlas.org). The results clearly demonstrated that Purinergic genes *P2RX7* and *PANX1* protein expression levels were significantly higher in tumor tissues than in normal kidney tissues (Figure 7G, 7H).

## DISCUSSION

The Purinergic pathway is associated with extracellular nucleotide and nucleotide receptors [8]. The earliest discovery of this pathway dates back to 1929, when Hungarian physiologists Szent-Gyorgyi and Drury found that injections of purified adenine temporarily reduced heart rate in animals, speculating that purines might have a role in transmitting extracellular signals. Burnstock et al. proposed the Purinergic neurotransmission hypothesis half a century later, describing ATP as a non-adrenergic and non-cholinergic neurotransmitter [9, 42]. Subsequent studies have further confirmed the validity of this hypothesis that extracellular nucleotides and adenosine can regulate cell proliferation, apoptosis, and other physiological processes by interacting with specific receptors [43, 44].

In this study, we selected 33 genes closely related to the Purinergic pathway and performed a pan-cancer analysis of these genes. The results of the research showed that these Purinergic genes were differentially expressed in various cancers, and the degree of gene methylation could impact the survival risk of tumor patients. These results suggest that the Purinergic pathway may be a very critical factor in the development of tumorigenesis. We tried to classify these Purinergic genes roughly into four classes according to the Purinergic receptor classification: P2RX, P2RY, P1, and others [42].

P2RX-like receptors consist of seven subtypes (P2RX1-P2RX7) that regulate biological processes such as tumor cell proliferation, apoptosis, migration, and invasion, mainly through ion channel-mediated signaling [45, 46]. It has been shown that overexpression of P2RX7 can promote colorectal tumorigenesis and assess the prognosis of colorectal tumor patients [47]. Also, P2RX7 can regulate the expression of the cell adhesion molecule E-calmodulin through the AKT signaling pathway, thereby promoting the growth and migration of breast tumor cells [48]. In addition, high levels of ATP can also promote apoptosis through the P2RX7-PI3K/AKT signaling pathway [49]. Based on existing research, we hypothesize that P2RX1, a P2RX-like receptor, is one of the Purinergic genes closely related to KIRC development. Still, no studies investigated the relationship between the two. However, some findings have shown that overexpression of the P2RX1 gene is strongly associated with disease progression and shorter survival time in urological malignant bladder cancer. The P2RX1 gene’s high expression increases the risk of distant metastasis of bladder cancer cells [50].

The P2RY class of genes is a member of the G protein-coupled receptor family that can regulate intracellular signaling by interacting with G proteins and has eight isoforms, including P2RY2, P2RY6, and P2RY11 [51]. P2RY2, P2RY6, and P2RY11 are three Purinergic genes that we believe are closely related to KIRC but are currently less studied about kidney cancer. P2RY2 is significantly expressed in prostate tumors, a cancer of the urinary tract, and inhibition of P2RY2 expression affects the epithelial-mesenchymal transition process of tumor cells and the expression of invasion-related genes, which subsequently affect the invasion and migration of tumor cells [52, 53]. In addition, similar results were shown in mammary tumor experiments at P2RY2, indicating that P2RY2 most likely regulates tumor cells' migration and invasion process by regulating the expression of cell adhesion-related genes Snail and E-calmodulin [54]. In contrast, P2RY6 was found to increase the resistance of tumor cells to chemical drugs in colorectal tumors, which is closely related to P2RY6 blocking the apoptotic process and contributing to the persistent development of colorectal tumor cells [55]. In addition, excessive ATP production during tumorigenesis can disrupt the autocrine feedback mechanism mediated by P2RY11, leading to defects in T cell migration and function, affecting host immune defenses, and negatively impacting tumor patients [56]. The Purinergic gene GNAS is also a member of the G protein-coupled receptor family, which is closely associated with the development of KIRC, but does not belong to the P2RY class [57, 58]. It has been shown that GNAS has activating mutations in most of the KIRC, and overexpression of this gene in KIRC may act as a promoter of tumor cells through a PKA-dependent pathway [59, 60]. In addition, in previous studies, this gene has been shown to predict survival in KIRC patients, providing new evidence for the observation of disease progression [61].

The P1 class of receptors is also an important component of the Purinergic genes and is implicated in the progression of KIRC. The P1 class includes four isoforms, including ADORA1, ADORA2B, and others. These Purinergic genes also belong to the G protein-coupled receptor family, which is adenosine selective [62, 63]. Several studies have shown that these Purinergic genes are aberrantly expressed in various cancers, such as thyroid and lung cancer, and can be used as potential diagnostic and prognostic markers, consistent with our findings [64, 65]. Also, this class of Purinergic genes positively correlates with the expression of various immunomodulatory factors during tumor development and promotes the immune escape of tumor cells through the ATF3-PD-L1 signaling pathway [64, 66].

The differential effects of Purinergic genes in various tumors have attracted our focused attention. According to our findings, most Purinergic genes play the role of risk genes in KIRC, and they are expressed at significantly higher levels in KIRC tissues than in normal kidney tissues. To assess the expression of Purinergic genes in KIRC samples, we used the mRNA expression levels of Purinergic genes for scoring. We divided the KIRC data samples into three groups based on this score. Subsequently, we analyzed the survival curves of these three groups of samples. Surprisingly, our results showed that the survival rate of KIRC patients in the Purinergic gene low expression group was significantly lower than that of patients in the Purinergic gene high or normal expression group. This finding contradicts our previous conclusion that most Purinergic genes play the role of risk genes in KIRC.

To explain this anomaly and to elucidate the specific mechanisms of Purinergic genes in the development of KIRC, we delved into the correlation between the expression levels of Purinergic genes and the expression of histone modifier genes as well as classical oncogenes. Our results showed that in the Purinergic gene low expression group, the expression levels of histone modifier genes (such as SIRT and HDAC) as well as classical oncogenes had highly significant differences compared to the other two groups (Purinergic gene high expression group and Purinergic gene normal expression group). In addition, previous studies have demonstrated that increased HDAC activity promotes the epithelial mesenchymal transition (EMT) process and enhances the invasion and metastasis of tumor cells [67]. Meanwhile, several studies have also revealed that tumorigenesis-related signaling pathways, such as PI3K/Akt, Wnt/β-catenin, and NF-κB, are closely related to the activity of SIRT, and that alterations in SIRT activity may affect the activation pathways of these pathways, thereby promoting the process of tumor cellular development [68–70].

In addition, mutations in classical oncogenes such as MYC, TP53, and KRAS cause tumor cells to evade cell cycle regulation and apoptotic signaling, which in turn affects the progression of programmed cell death in tumor cells, resulting in continued disease progression [71–73]. It deserves special attention that classical oncogenes are closely related to the expression of tumor antigens, and changes in the expression level of classical oncogenes affect the expression of tumor antigens in the tumor microenvironment. This may result in the inability of dendritic cells to recognize presented tumor antigens, leading to immune escape situations [74, 75]. In targeted tumor therapy, classical oncogenes also play a role as tumor drug targeting sites, and changes in the expression of classical oncogenes can affect the action of tumor-targeting medications, leading to drug insensitivity or resistance in tumor cells [74, 76] These findings explain, to some extent, the lower survival rate of KIRC patients in the Purinergic gene low expression group and provide new ideas to explore the mechanism of the role of the Purinergic pathway in KIRC.

In exploring the effect of Purinergic genes in tumors, we noticed that these genes play an important role in tumor immune processes. To clarify the connection, we performed an immune infiltration analysis of Purinergic. The results showed a high positive correlation between the Purinergic pathway and the processes of Parainflammation, CCR, and T-cell co-stimulation in the immune response. Previous data suggest that multiple CCRs (CCR3, CCR5, CCR6) are overexpressed in KIRC and can regulate T-cell cytotoxicity and antigen presentation by dendritic cells. This activity contributes to creating an immunosuppressive environment, which in turn promotes the development of tumor cells [77–79]. Meanwhile, the co-stimulatory molecule CD28 can amplify tumor-specific cytotoxic T lymphocytes during the development of KIRC. This phenomenon may be closely related to the drive of B7-1/CD28 co-stimulation of IL-2 [80, 81]. These findings confirm the close relationship between the development of KIRC and these immune-related factors. Targeted interventions targeting the Purinergic pathway could impact these immune-related factors and provide new ideas for the immunotherapy of KIRC patients.

With advanced knowledge of disease biology, current first-line treatment options such as targeted therapy and immunotherapy for KIRC are widely used [82]. The U.S. Food and Drug Administration (FDA) has approved targeted drugs such as Sunitinib, Pazopanib, and Axitinib for treating KIRC [83]. Studies have confirmed that Sunitinib and Pazopanib have good progression-free survival in the first-line treatment of KIRC but may cause serious adverse effects and lead to some drug resistance. In contrast, Axitinib, a second-generation drug, improves efficacy while reducing the incidence of adverse reactions and, therefore, may be a second-line treatment option for KIRC [84]. However, some studies have found that targeted agents do not have the desired effect in some KIRC patients, that tumor progression is not significantly inhibited after treatment, or that tumor cells resist these targeted agents [85]. It may be due to the differences in gene expression in tumor cells causing changes in EMT processes, epigenetic modification processes, and tumor microenvironment factors. These changes could have an impact on the mechanism of drug resistance in tumor cells [86].

We used the Purinergic score in the above study to determine the mRNA expression levels of Purinergic genes in different KIRC samples. We divided the KIRC samples into three groups accordingly. We analyzed the IC50 of targeted drugs in these three groups, and the results showed significant differences in sensitivity to targeted drugs in the different groups of patients. This finding suggests that we can individualize the selection of different targeted drugs for precise intervention according to the Purinergic gene expression level of KIRC patients. With this individualized treatment strategy, KIRC patients can achieve better treatment outcomes and reduce the occurrence of side effects, thus improving the overall effectiveness of treatment.

By LASSO regression analysis, we selected nine Purinergic genes (P2RY8, P2RX1, GNAS, P2RY11, ADORA2B, PANX1, ADORA1, P2RY6, and P2RY2) to construct a KIRC survival model. By performing ROC curve analysis on the model, we observed high AUC values, indicating that the survival model has good predictive ability and can be used to predict the survival of KIRC patients in the next 5, 7, and 10 years. Also, through univariate and multifactorial regression analysis, we identified risk score as one of the independent risk factors affecting the prognosis of KIRC patients. To conveniently apply this survival model for prognostic assessment, we further constructed a nomogram that provides clinicians with an intuitive tool to accurately determine the predictive risk of patients based on their risk scores and other relevant factors, thus guiding individualized treatment plan development.

Of course, there are still some limitations to our study. In this study, we only analyzed the possible mechanism of action of the Purinergic pathway in KIRC from a bioinformatics perspective. We did not elaborate on the specific mechanism of action of the Purinergic pathway in KIRC, which may require more refined preclinical experiments to reveal the connection, which is the main direction of our future research. At the same time, we used retrospective data from public databases. The data quality may be affected by sequencing technology, sample processing, data algorithms, and statistical methods, and the biological heterogeneity between samples may also affect the results of replicated experiments. In addition, translating research results into clinical applications is a great challenge, and we need to collect more clinical data from real-world KIRC patients to optimize the accuracy of the model in the future to ensure that the model can provide the most accurate predictive function for clinicians and benefit more KIRC patients.

In conclusion, our Purinergic gene-based KIRC survival model has high predictive accuracy. Although there are still some limitations, the model can provide important support and guidance for clinical research and individualized treatment and new ideas to improve prognostic assessment and treatment strategies for patients with KIRC.
